# Theory of Sensation

**Published:** 1811-11

**Authors:** 


### Medical and Physical Journal.

VOL. XXVI.] NOVEMBER, 1811. [NO. 153.

Printed for R. PHILLIPS, by E. Hemsted, Great New Street, Fetter Lane, London.

### *For the Medical and Physical Journal.*

### *Theory of Sensation.*

(Continued from P. 105 of this Vol.)

OF THE ACTION OF VESSELS IN INFLAMMATION.

TWO different opinions respecting the state of vascular action in inflamed parts appear to exist at the present day; according to the one the action of the small vessels being increased, and according to the other diminished. As the former is of no late date, and is still perhaps most generally adopted, it cannot be necessary for me to state the arguments that have been used in its support. The other opinion, viz. that the action of the capillaries of an inflamed part is diminished, was said to have been first advanced by Dr. Lubbock and Mr. Allan. I have not, however, seen any statement by either of these Gentlemen of the facts which led them to embrace this opinion. I must therefore refer the reader to a paper of Dr. Wilson's inserted in the Edin. Med. and Surg. Journ.*, in which may be found the reasoning and experiments which induced him to adopt the same opinion. These experiments, which appear to have been conducted with great care and accuracy, are highly interesting, and shew in a very satisfactory manner that the velocity of the blood becomes less and less as inflammation increases, and that at the acme of inflammation the circulation appears to cease altogether.

Without wishing in the least to invalidate the Theory Dr. Wilson has erected upon these experiments, it is proper to remark, that however favorable at first view they may appear

* Vol. IV. p. 290.

pear to the principle of Sensation I have ventured to adopt; still I have not been able to make any use of them in my inquiry; not only because they were all performed on brute animals, in whom we cannot but by vague conjecture judge of the state of the sensation, but because that Gentleman, if I am not mistaken, has entirely overlooked some very important actions, particularly the formation of new vessels, which there is no cause to doubt often takes place in inflamed parts, at the very time the circulation of the blood is in that state of diminished velocity or apparent stagnation, so clearly and satisfactorily evinced in his experiments.

In the course of this investigation I endeavoured to ascertain singly and independent of each other the state of the circulation, secretion, excretion, absorption, and of those actions by which new vessels are formed or old ones enlarged, in inflamed parts. I shall, however, confine myself in this paper to the consideration of the first and the last of these actions; both for the sake of conciseness, and because these appear to be most strikingly affected in inflammation, and to have the closest connection with the pain.

The state of the circulation will hereafter appear, I hope, to be of so much consequence to the actions of formation and repair in inflamed parts, that although I do not consider diminished velocity of the blood to be the cause of the pain in inflammation; yet I may be excused for giving a short account here of the experiments by which I endeavoured to ascertain whether the motion of the blood is quicker or slower than natural in inflamed parts.

### 1. Of the state of the circulation of the blood in parts inflamed.

That the small vessels in their healthy state assist in propelling the blood thrown into them by the *vis a tergo* is now, I believe, generally acknowledged. That they possess some degree of action of this kind is obvious from this fact, among others, that if the arteries leading into a part be compressed so as to prevent the influx of blood into the part, it immediately becomes more pale, a proof that the small vessels contract themselves and propel their contents.

### *Experiment* I.

The back of my hand being somewhat red, but without any sensation in it, I held my fore arm and hand in an horizontal position, and compressed the humeral artery at the bend of the arm so as to stop the pulse of the wrist, taking care at the same time not to stop the reflux of the blood in in the subcutaneous veins. A very sensible diminution of redness in the back of my hand immediately took place. On repeating this experiment at different times and in different persons, I found that the paleness is not always equally produced. Perhaps this may be owing to the influx of the blood by the artery being less completely stopped, or the reflux by the veins more obstructed in some trials than in others. In the following experiment the contractile power of the small vessels is better evinced.

*Experiment* II.

I tied a ligature firmly around the fleshy part of my fore-arm. This prevented the return of the blood by the subcutaneous veins, but did not stop the pulse at the wrist. The hand soon became very red, as if inflamed. I now compressed the humeral artery above the bend of the arm, to prevent the reflux of new blood; and caused the ligature to be untied. This hand soon became as pale as the other.

These experiments shew that a contractile action of the small vessels does exist; and are used as tests in some of those which follow.

*Experiment* III.

To ascertain if the velocity of the blood was greater or less than natural, I immersed my hand in very hot water, and having suffered the pain, which was excessive, as long as possible; on taking it out I found it highly inflamed and somewhat swelled. I now compressed the humeral artery above the bend of the arm. The redness and turgescence continued for some minutes. The pain went off first.

*Experiment* IV.

A lady had an inflammation and swelling in the back of two of her fingers attended with throbbing pain. I compressed the humeral artery as above. The blood immediately forsook the rest of the hand, which assumed a death-like paleness. But as long as I compressed the artery, which was upwards of a minute, the inflamed parts continued as red as before. When the artery was compressed the throbbing ceased, but a smarting pain remained.

*Experiment* V.

A gentleman had an attack of gout in the first joint of the thumb of one of his hands. It was highly inflamed, tense, and he felt a gnawing, burning pain in it. I carefully compressed the humeral artery above the bend of the arm. The gnawing pain ceased, but the redness continued undiminished.

These experiments correspond in effect with those of Dr. Wilson, and prove that the velocity of the blood in inflamed parts, instead of being greater as was formerly supposed, is actually much less than natural. For if the action of the small vessels by which the blood is propelled in them had been so much increased, as considerably, or at all to augment the *velocity* of the blood; the inflamed parts in these experiments ought to have become pale sooner than natural; the reverse of which in these and numerous other trials I have made has been uniformly found to take place.

But although the *velocity* of the blood in the small vessels of an inflamed part is greatly diminished, it does not follow as a direct consequence, that the *action* of these vessels is diminished also: for it has not yet been proved that as much blood is not propelled in a given time by the action of the vessels of an inflamed, as of a sound part; and that the redness continuing so much longer in the former than in the latter, after the accession of new blood is cut off by compression of the artery, is not entirely owing to the difference in the quantity of blood required to be moved.

Thus if we suppose the power by which the small vessels propel the blood in them to be as 1, and the quantity of blood they contain in their healthy state also as 1, and the quantity they contain in a state of inflammation as 5 or 10 or more; then it is apparent, that the moving power of the vessels being in both cases the same, they must take as many more times to empty themselves in an inflamed state, after the influx of blood is prevented, as the quantity of blood contained in them exceeds the natural standard.

### *Experiment* VI.

The subject of this experiment was the same as that of the fourth. It was there mentioned that as soon as the humeral artery was compressed the throbbing pain ceased. It returned, however, and the rest of the hand regained its natural colour immediately after the pressure was discontinued. I now resolved to observe with accuracy the time which the vessels of the inflamed parts took to empty themselves, compared with that in which the uninflamed parts became colourless. I compressed the humeral artery as before. The uninflamed parts of the hand had changed from their natural healthy appearance to a remarkable paleness, in three or four seconds at most. The colour of the inflamed parts was not sensibly changed at the end of a minute, and a smarting pain remained.

remained. In the course of the second minute the pain went off, but the redness continued apparently the same. At the end of the third minute the inflamed parts were sensibly collapsed, and had changed from a scarlet red to a pale brown colour. I now desisted from pressing the artery. Neither the pain nor redness returned, and the inflamed parts resumed their natural appearance.

If the quantity of blood circulating in the inflamed and uninflamed parts, in this case, was as 15 in the former, to 1 in the latter, (which is probably more than the maximum in that degree of inflammation,) then the red colour of the inflamed parts ought to have disappeared at the end of a minute, or sooner, if the moving power was as great as natural. But instead of this no sensible decrease of the redness was perceived even at the end of two minutes; and it was principally in the course of the third minute that the change of colour and collapse, apparent at the end of that time, must have been effected. The action of the blood-vessels of the inflamed parts, therefore, was greatly diminished, unless they contained more blood than the proportion supposed, which is not probable.

But as these proportions are only assumed, it was desirable to substitute some other mode of judging in which nothing should be left to conjecture. I therefore thought of the following method by which the quantity of blood in the inflamed and uninflamed vessels is made to be exactly the same.

*Experiment* VII.

I wound a piece of small twine spirally around the upper part of the third phalanx of one of my fingers, so firmly as to prevent, very effectually, the blood from returning by the veins, but not to hinder the blood by the arteries from entering the finger below the ligature. The finger soon became turgid and very red. When these effects had taken place, I firmly compressed the artery above the bend of the arm; the pulse at the wrist ceased. I then caused the ligature to be removed, and observed the time which the vessels took to empty themselves afterwards, still carefully preventing the influx of new blood by the arteries. In somewhat less than thirty seconds this finger was as pale as the others.

*Experiment* VIII.

I again twisted a piece of twine, in the same manner and with the same degree of firmness as before, around the finger, and when it appeared as red and turgid as in the last experiment, I compressed the humeral artery, as above, with the effect effect of stopping the pulse, and immersed the finger in water so hot as to induce violent pain, which I suffered for about a quarter of a minute. I now took it out of the water and caused the ligature to be removed, still carefully compressing the artery. In half a minute after the ligature was untied (the time in which the finger became pale in the seventh experiment) the finger appeared as red as before; at the end of a minute it appeared the same; at the end of a minute and an half part of the finger had regained its natural colour, and at the expiration of two minutes there was still a considerable redness of the skin over the knuckles, which did not appear in the other fingers. The pain went off before the redness.

The very great difference of time which the vessels in these two experiments took to empty themselves of *the same quantity of blood*, proves, I think, beyond a possibility of mistake, that the contractile power of the small vessels is diminished during inflammation, or that it is exerted with diminished effect.

With regard to the pain.—From these two experiments, as well as from the fifth and sixth, and numerous other experiments and observations of the same nature, which I have made, I infer, that the pain in inflammation is not caused by the diminished motion of the blood, or diminished action of the blood-vessels containing it. Because in the seventh experiment, in which the motion of the blood was prevented by a mechanical cause, and in which, of course, the contractile power of the vessels was exerted without effect, a sensation of weight or fullness only was felt, very different from inflammatory pain; and because not the least pain sometimes is discernable in inflamed parts, in which that impaired state of the circulation obtains, as at the end of the fifth and sixth experiments.

Is there, as was supposed by Mr. Hunter, an action of *dilatation* rather than of *contraction* in the small vessels of an inflamed part? This is a question of importance; and any Theory of Inflammation that shuns it entirely, must in that respect appear defective. I will, however, defer this question at present, and resume it after examining the state of those actions by which vessels are formed, &c. when it will fall more properly to be considered.

With regard to the larger arteries leading into an inflamed part, their action does not appear to form an essential part of this inquiry, because, though generally allowed to be increased, no part of the pain is referred to them.

### 2. Of the plastic actions in inflamed parts.

The operations connected with the present inquiry, to which this term appears not unappropriate, and which for that reason, and to avoid circumlocution, I shall take the liberty of using occasionally when speaking of these actions, are,

1st. Those by which organizeable fluid is separated from the blood, and new vessels made to shoot into it, as in adhesion, granulation, enlargement of vessels, &c.

2d. Those by which the organization is adapted to the secretion of pus.

3d. Those by which the organization is adapted to morbid secretions and supported when formed, as in ulcers not purulent.

4th. Those by which organized parts injured, without apparent loss of substance or texture by mechanical or other causes, are repaired.

5th. Those by which new and peculiar actions and consequent organization, excited by internal causes, are erected on particular structures, as in gout, erysipelas, scarlatina, small-pox, &c.

All the operations here mentioned appear to be strictly *plastic;* because none of them seem to exist previously, at least in equal degree, till the stimulus is applied. Thus, when a wound with a sharp cutting instrument is received, a change of action takes place in the wounded part either in kind or degree, the organization becomes adapted to the circumstances; if the edges of the wound are in contact or nearly so, coagulable lymph is in general separated from the blood; new vessels shoot into it from both sides of the wound, and the continuity of the part is restored. But when there is a loss of substance in the wounded part, and especially when its edges are lacerated and contused, a suppuration is in general necessary: but before pus can be secreted, the organization must be rendered fit for its secretion; for this purpose new vessels must be formed, and when formed must be supported: for no such action or organization adapted to it has ever been supposed to exist in sound parts. The same is true of ulcers, and, probably, may be found to be equally so in gout, scarlatina, &c. at least no harm, I hope, can result from considering it so till the nature of the actions in these diseases is better understood. Mr. Locke justly observes, that "the way to improve our knowledge, is to get, and fix in our minds, clear, distinct, and complete ideas, as far as they are to be had, and annex to them proper and constant names." In using the words *gouty action, inflammatory action*, &c. it is surely necessary to attach some settled meaning to the terms, and the above comprehends the most of, or all the actions which appear to be understood to prevail in the different species of inflammation. I shall now, therefore, proceed to shew by cases what has appeared to me, after a very strict attention to the subject, to be the connexion between these actions and the pain. The cases are not detailed as presenting any thing singular in occurrence, or novel in practice. They are selected from many more of the same kind, which it would be both tedious and useless to relate, solely with a view to illustrate what, I hope, will appear not an uninteresting subject.

Case 1.—A young man, in leaping from a height, fell and struck the outside of one of his thighs in descending against the sharp corner of a large stone; by which the integuments and adipose substance were torn for upwards of nine inches. I saw him soon after, and found that the wound had bled little, that the facia lata of the thigh was exposed by the retraction of the skin and adipose substance, and that the edges of the wound were nearly as smooth as if it had been made by a sharp cutting instrument. The wound appearing perfectly clean, I brought the edges of the divided integuments together by means of the interrupted suture and adhesive straps, supporting the whole with a bandage, and enjoined the patient to avoid moving the thigh. I saw him again next day, and being informed he had felt no pain in the wound from the time it was dressed, I removed one of the adhesive straps in order to ascertain if reunion had commenced. This had so far taken place that I could not separate the edges by gently pulling the skin on each side of the wound in opposite directions. This gave some pain, however, showing perhaps with still more certainty that the bond of union had become vascular or organized. It is unnecessary to lengthen the case: suffice it to say, that the cure went on without interruption, and *without pain*. From the very commencement of this case, a blush of redness surrounded the edges of the wound, which continued long after the continuity was perfectly restored; the vessels, therefore, of that part, must have suffered either a forcible or spontaneous dilatation.

Case 2.—A man wounded himself with an axe in his right leg. The cut was longitudinal, on the outside of the tibia, free of the bone, and was about six inches in length, but not very deep. It bled moderately. There was a slight degree of redness around its edges, but the patient was free from pain. The edges of the wound were brought into contact, and retained by the same means as in the preceding case; and the patient was ordered, when out of bed, to keep the foot and leg on a level with the knee. I did not see him again till the second day after. He complained that the wound had given him great pain, and begged I would remove the bandages, bandages, as he thought the pain was caused by them. I suspected that he had been walking with it. He confessed he had, and that the pain came on afterwards. On removing the straps I found the edges tense and inflamed, but no adhesion had taken place. A smart cathartic was now ordered him, and he was strictly enjoined to keep the leg as much as possible in an horizontal position; in hope that if by these means the pain was removed, suppuration might be prevented, and union by the first intention still obtained. On visiting the patient next day I was informed that the medicine had operated quickly, and that he had felt no pain in the wound since that time (about eighteen hours). I now removed the straps cautiously, and had the satisfaction to observe, that the adhesive process had evidently begun, as the edges of the wound appeared as if glued together, and were not separable by a gentle force. Nothing further occurred worth mentioning, but that he felt no pain from this time, and in a few days could use the leg as usual.

Case 3.—A man received a cut with a knife in the palm of his hand. I was not called to him till the fourth day after the accident. The whole hand was then inflamed and painful. The wound, which extended across the ball of the thumb into the palm was full of black hardened blood. Its edges were livid; and the pain had been, and still continued so severe, as to prevent sleep and induce considerable fever. An emollient cataplasm was ordered to be applied immediately to the wound, and to be renewed frequently. Next day the patient informed me, that soon after the first poultice was applied he felt a great relief from the pain; that when the dressings were changed, some black lumps mixed with a dirty looking matter came away; and that last night, for the first time since the accident, he had enjoyed several hours sleep. I found him now more free of fever; and on removing the dressing the hand appeared less inflamed: the wound was almost clean, but appear'd much larger than the day before; and it was discharging matter copiously, which, though thin, was puriform. Soon after this the purulent discharge became less abundant, and of a better consistence; the wound gradually filled up with healthy granulations; the edges approached nearer and nearer to each other and at length coalesced; and during the whole time of the process the patient enjoyed a complete immunity from pain.

It cannot be necessary to multiply cases of this kind. They are sufficiently common; and the relation between the *pain* and *action* in every case I have seen was exactly the same. That relation is sufficiently striking. In the first case the adhesive process went on without interruption and no pain was felt. In the second case the same actions were interrupted for some time; pain was felt during that time, but no longer; for, apparently, as soon as the adhesion was allowed to go on without resistance, sensation ceased.

But might not the pain in the second case arise from a change of action? Might not the organization at the time be changed and adapted to the secretion of pus? If this had been the cause of the pain, suppuration ought to have preceded the healing of the cut; which it did not. In many such cases, the adhesive process is by some cause prevented, considerable pain is felt, but by removing the pain by proper means, suppuration, which would otherwise supervene, is prevented, and pure adhesion takes place. It seems, therefore, most probable, that the pain is owing in the first instance to the interruption of the adhesive process. But when the interruption to this process has gone to a certain extent, union by the first intention becomes impracticable, and suppurative action is probably next attempted. In the third case, however, suppuration was attempted, adhesion being interrupted, it is obvious that this change of action also was prevented, for the edges of the wound were livid, and tending to gangrene. Hence, it would appear, that the pain in that case arose in the first instance from interruption to the adhesive process, its increase and the increasing inflammation, from the suppurative process being interrupted; and in the last instance, it may appear to be connected with interruption to the ulcerative process, by which gangrenous parts are separated and thrown off. This process at the end of the third case became necessary, but when it came to be performed without interruption, the pain ceased.

A remarkable circumstance attending the plastic actions is, that when any cause of resistance appears to obstruct their performance, in the part where they are attempted, the sensibility or irritability of the nerves of that part is very generally, if not universally increased. Does this arise from the plastic actions, interrupted in vascular parts, being transferred to the nerves? If so, it might be expected that the size of the nerves of an inflamed part would be augmented thereby. But this is said not to be the case.* The plastic actions, therefore, if transferred to the nerves, appear to be also interrupted there. If enlargement of the nerves, proportioned to the degree of plastic action interrupted in vascular parts, took place, would this increased sensibility or irritability occur? I know, at present, of one case only which, if

* Hunter on Inflammation, p. 288.

I mistake

I mistake not, bears strongly on this question, a case of jaundice, occasioned by the pressure of a large hydatid in the liver, by Dr. Duncan, Sen. Professor of the Institutes of Medicine in the University of Edinburgh.* In this highly interesting, and minutely detailed case, although the obstruction to the passage of the bile through the ducts was in some measure compensated for by increased absorption producing jaundice; yet from the dilatation the uncompressed part of the ductus communis next the liver had undergone, it seems evident that the absorption had not been always equal to the secretion of bile. But the bile accumulating in such quantity as to dilate the duct, must finally, I suspect, have been attended with the effect of interrupting, in some measure, the further secretion of bile, till the absorbents have removed the superabundant quantity. Here there was obstruction to the *vascular* actions of the liver. But the *plastic* actions appear to have been increased; for the liver, although free of disease in the glandular part, was of uncommon bulk, "the hepatic nerves were much larger and much harder than common," and "the branches of the hepatic nerves could be distinctly traced along the gall ducts. The adjacent parts betrayed marks of previous inflammation. The stomach and great arch of the colon were more intimately united than usual by the omentum: and there was also a strong adhesion between the liver and the kidneys." Yet the patient in this case had expressed very little pain, incomparably less than (from the enlarged appearance of the nerves, liver, &c. the undoubted consequences of increased plastic action) might have been expected, if increased action were the cause of the pain in inflammation. Nor does there appear to have been any considerable increase of irritability or sensibility in this case, certainly not any increase equal to that which takes place in inflammations, in which no augmentation of the size of the nerves is perceivable. It would be unjustifiable to draw any general conclusions from a single case. I will therefore not attempt it. I cannot, however, forbear remarking here, that if cases were always taken down with the same accurate attention to the *sensations* as well as to the functions in disease, and if dissections were performed and related with the same minuteness and precision, as in the case above referred to; considerable light might perhaps at length be thrown on certain interesting functions of the animal economy, which hitherto have escaped detection, or eluded research.

But may not the increase of irritability that occurs in in-

---

* See Edin. Med. and Surg. Journ. vol. 4. p. 187.

flamed parts be owing to an increase of *nervous* action, or accumulation of an active principle in the extremity of the nerves, in consequence of the plastic action attempted, being prevented? This question I shall not pretend to determine. It is proper, however, to observe, that if this increased irritability is owing to an increased action in the nerves different from augmentation of size, that increased action is not the cause of the pain, because in many cases of inflammation, the irritability or sensibility is exquisitely increased, without, however, any pain being felt, except on the part being pressed, &c.

The two following cases, though not belonging to the order of phlegmasiæ, shew at least the connection between the plastic actions and the pain.

Case 4.—A woman, about 70 years of age, came under my care with an ulcer on the forehead, which had remained several years, notwithstanding numerous remedies had been applied. It was nearly circular, about the size of a crown piece. The edges were slightly elevated and hard. It discharged a thin sanies which did not appear acrid or eroding, as no considerable enlargement of the ulcer had taken place for above a twelvemonth. Occasional pains, but very slight, were felt shooting through it, and hence (perhaps prematurely) it had been pronounced to be cancerous. I gave her two drachms of Fowler's solution diluted with an equal quantity of water, with orders to bathe the ulcer night and morning with a little of it, and afterwards to cover it with a pledget of lint. In about eight days she called on me again for a new supply of the solution. She informed me, that for a minute or two after the three or four first applications of the solution, she had felt a considerable smarting pain, but that it now produced only an agreeable glow; and that the shooting pains had entirely ceased. On looking at the ulcer I found that it appeared quite healthy, was filling up with granulations, and covered with pus of a proper consistence. The edges too had subsided, and the diameter of the sore appeared to be less. I repeated the solution, but desired her not to apply it again unless the pain should return. As the patient lived at some distance I did not see her again till about a fortnight after this. She had not needed again to apply the solution. The ulcer now was quite superficial, and not above a fourth part of its original diameter. Soon after this it healed without any further trouble.

In every ulcer, which, like the above, is attended with a considerable discharge, not purulent, with little or no pain, it may, perhaps, be taken for granted, that a morbid association of actions has superseded the healthy actions of the part; and that before the ulcer can heal, with a probability of remaining whole, the discharge must not merely be diminished, but the morbid association must be dissevered and destroyed in its whole extent. Applications which give pain, but do not enlarge the ulcer, appear, in the first instance, to obstruct the actions going on at the time. The effect of this interruption will be, that the efforts to restore that action will be increased, or these efforts being unsuccessful, a new mode of action will be attempted. In the above case the morbid association of actions, interrupted by the applications of the arsenical solution, appear to have been completely broken, and exchanged for salutary plastic actions; because, notwithstanding the age of the patient, and the long time the ulcer had continued, the cure was permanent. The ceasing of the pain after the plastic actions were evidently performed with success, deserves notice.

Case 5.—This also was a case of ulcer in the forehead. It was about two inches in breadth, and four in length, extending from the left eye-brow to above an inch within the hairy scalp. Its edges were hard and retorted, and extremely tender when touched: and the feeling of the skin around was somewhat benumbed. The bottom of the ulcer was covered with a white slough. It discharged a fœtid sanies. The subject of this case was a woman between 70 and 80 years of age. It had commenced about fifteen years before. Numerous applications had been tried both by regular and irregular practitioners to whom she had applied, but without benefit, as it continued to spread gradually. Its progress for a week or two before I saw her had been uncommonly rapid. This she imputed to some applications which had given her great pain. Frequent severe lancinating pains were felt in it. During the night, especially, the pain was violent, extended over the greater part of the head, and prevented her from enjoying any sleep till towards morning. Her health was otherwise good.

This appeared a very hopeless case. However, I gave the arsenical solution a trial. But as it gave great pain, increased the discharge, and enlarged the ulcer rapidly, without appearing to alter its nature, it was abandoned in a few days. I now gave her some carbonas magnesiæ to strew over the ulcer to protect it from the air, and also with a view to prevent it from spreading, by taking up the acrid matter as fast as it was secreted. I was not prepared to expect the effects that followed. The powder was applied three times a day, the ulcer being bathed with milk and water before each application. The pain became less and less every day, and before the end of a week the patient was entirely free of it. In the mean time the bottom of the ulcer became clean, and began to be covered with apparently healthy granulations, attended tended by a discharge of pus. So rapidly did these granulations advance, that in less than a fortnight the ulcer was closed, except a small part above the eye-brow. There the edges remained hard and retorted, extremely tender to the touch, and the surrounding skin benumbed. When the granulations had risen to a level with the integuments, a skin was formed over them in little more than one night. After this a remarkable change took place. The pain immediately recurred with uncommon violence. The blood seemed to forsake the new formed parts, and the ulcer appeared as if filled with dough. The pain continued day and night with but little remission, till the new formed parts were converted into a white slough, such as appeared at the bottom of the ulcer when I first saw it: the discharge then returned, the ulcer appeared as large as ever, and the pain abated to resume its accustomed periods of recurrence.

In this case the absence of the pain and of the morbid secretion while the plastic actions were going on is remarkable; and the return of the pains immediately after these were almost completed, is no less so. This may be ascribed to a return of the morbid actions. But the form in which the morbid actions appeared before was that of secretion. If this secretion was attempted immediately after the plastic actions were nearly finished, it was not performed with success; perhaps the new formed vessels were an obstacle in its way, for the secretion did not appear till the slough was formed. The pain therefore may with as much probability be ascribed to the resistance this action met with, especially as it abated after the secretion was renewed. But might it not be also partly owing to the efforts to preserve the new formed organization? This case shows, however, that when action is performed without resistance no pain is felt; and that even morbid actions produce pain only as they are interrupted, or interrupt some other action. The same appears to happen with other morbid actions. Thus, in the medullary sarcoma, or fungus hæmatodes, the growth and enlargement of the tumour has in some instances produced no pain. And when pain is felt, it is rather in such circumstances as the growth of the fungus might be supposed to be most resisted; or which, perhaps, amounts to the same thing, in which the surrounding parts do not spontaneously recede but are forcibly stretched, before the increasing tumour. In this case it seems obvious that the morbid actions will be interrupted in the ratio of the difficulty with which the parts give way to them. The fact that this interruption commonly proves a stimulus to greater efforts, does not seem to affect the truth of this conclusion. Whatever be the force of the efforts, the pain appears pears to increase or diminish according as they are more or less successful. In like manner when inflammation terminates in abscess, it is well known that as soon as the suppurative actions commence the pain commonly abates. But in some instances the parts containing the abscess yield with so much difficulty, that the quantity of pus secreted in a given time cannot be equal to the efforts to secrete. In these circumstances the pain, instead of abating after suppuration begins, sometimes increases. Thus in that species of whitloe in which the matter collects under the sheaths of the tendons or periosteum, the pain is excessive, even after suppuration has begun, and nothing gives so immediate ease as making a free incision down to the bone; which not only gives vent to the matter already accumulated, but allows the secretion afterwards to be poured out without interruption. In the Egyptian ophthalmia, when internal suppuration takes place, the pain is excruciating till the coats of the eye give way. The pain, in these cases, may be said to arise from the purulent matter collecting and pressing against the parts rendered morbidly sensible by the disease. But surely it ought not to be overlooked that this morbid sensibility, as before noticed, appears to be produced by interruption to vascular action, and that the morbid sensibility begins immediately to diminish when the resistance to vascular action is removed.

Case 6.—A young woman in running fell and struck one knee with violence against a stone. I saw her the following day. At this time the knee was highly inflamed, hot, tense, and exquisitely painful. The skin did not appear to have suffered much injury by the fall. Topical blood-letting was immediately used. Refrigerant lotions were afterwards kept constantly applied to subdue the painful sense of heat; and cathartics were administered to prevent effusion, or promote absorption, if effusion had already taken place into the joint. By these means the pain was removed, the redness gradually abated, and in two days nothing but a sense of weakness on moving the knee joint remained, which soon went off under the use of tepid bathing and gentle frictions.

Accidents similar to the above often happen, and the pain, though exquisite at first, gradually subsides and no further inconvenience is experienced. In such cases it will, I think, be granted without difficulty, that the organization deranged by the accident is almost immediately restored; in other words, the plastic actions are performed with little or no interruption. In the above case it seems next to certain, that if these actions had not been prevented the pain would not have continued. But a very constant effect of interruption to action and consequent pain appears to be, that it stimulates lates to increased *efforts*. If these increased efforts are unsuccessful the pain and inflammation increase; and when the *action* does come to be performed there is a risk that it may be performed in excess. In the above case, therefore, to remove the pain was not the only object in view. In the very painful state of the joint at the time I first saw the patient, it is almost certain that a mere removal of the pain would have been succeeded either by a copious effusion, suppuration, or a morbid thickening of the ligaments of the joints, all of which were to be guarded against. Relief from the pain, therefore, was to be obtained by means which diminished the congestion of fluids in the part, or promoted absorption. And the means employed appear to have been in a great measure successful. But that they prevented *future morbid actions* will not, I apprehend, appear to the mind of the intelligent reader any proof that their immediate effect was to *diminish action*. The primary restorative actions were doubtless performed after the pain ceased.

It may serve to illustrate this part of our subject to advert to a well known circumstance attending the treatment of burns. Where the organization of a part is considerably deranged, but not absolutely destroyed by fire; when it is still within a possibility of being repaired, by immersing the part in cold water, or applying cold substances to it and carefully preventing painful sensation, the injured part is preserved and soon restored to its sound state. But allow the pain to continue, and the actions of repair being interrupted, the consequence will be, that the inflammation of the part will increase, and the pain will not cease till either the injured parts sphacelate, or till an effusion of serum or suppuration come on. But would it be correct to assert that the cold applications in such a case as this *diminish action*. The result of the case is a proof that the *restoring actions* are not only performed, but best performed without pain. The first and second cases shew that adhesive actions are most successfully accomplished without pain. The third and succeeding cases evince that pain is not necessarily an attendant of suppurative action or of the formation of new vessels. But what other *vital* actions, that may account for the pain, take place in consequence of burning? If any other occurs it is yet undefined. I humbly conceive that no harm can result from distinguishing as far as possible in our practice as well as in our reasonings between *action* and mere *efforts* to act. As far as we have gone, the sensation, if I mistake not, indicates that the action falls short of the *effort*.

CASE 7.—A gentleman between 50 and 60 years of age, of an ingenious and eccentric turn of mind, and subject to frequent quent irregular attacks of the gout, to which he was liable both by inheritance and by his manner of living; after some dyspeptic and spasmodic affections, the usual precursors of a paroxysm of gout, was attacked one morning with a violent pain in his wrist and elbow joint, which were somewhat swelled, tense, and inflamed. In the course of the day the pain and inflammation increased, attended with a distressing sense of burning heat. He became sick at stomach, nauseated his food, and had occasional pains which he referred to the stomach, and which he said always seized him when the pain in the extremities was severe. For several years this gentleman (driven by instinct or desperation) had been in the habit of applying cold water to the inflamed joints till the pain abated, a practice which he had repeated often not only with impunity, but advantage, as he thought the paroxysm shortened by it, and his health otherwise improved. Unluckily, however, early one morning, in the height of a paroxysm, he got out of bed, and to procure water of sufficient coldness, went, in his shirt, to the pump in the garden, and began to pump the water upon the painful joints, but not with its usual happy effects. The pain, indeed, left the limb, but before he could return into the house he was seized with a violent pain about the scrobiculus cordis, which arrested him on the spot and prevented him from calling for assistance. He was therefore forced to lie down on the damp ground, and would probably have perished had not a servant discovered him by accident. He was immediately carried into the house, and by the assistance of warmth and cordials plentifully administered, he was at length, but not without difficulty, recovered. From this time the application of cold was totally abandoned; because he imputed its effects, in the last instance, to the influence of some imperceptible revolution that had taken place in his constitution.

I inquired if the immediate sensation produced in the inflamed joints by the cold water in the last and former instances was in no respect different. The only difference, he said, that he could recollect was, that formerly he had felt the cold water rather agreeable, but that in the last instance it was painfully cold, and *shot through him like an ice bolt.* I then suggested to him, that the bad effects which had followed the last application of cold, were probably less owing to any difference or change in his constitution, than to that difference in his sensations, which was very well accounted for by the manner and circumstances of the application. And that even in the present paroxysm, if he applied the water no colder and continued it no longer than relieved the pain and was grateful to his sensation, it might still be attended with its former good effects. As he did not think this suggestion improbable, and as the criterion of safety was then made to rest on his own sensations, he willingly consented to give the cold water another trial. Cloths wet with cold water were applied to the wrist and elbow joint, and were renewed as the returning sense of heat seemed to demand. This proved highly agreeable to his sensations, and he persisted with alacrity. Before I left him he expressed himself greatly relieved of the sickness and pains at stomach. He had taken no cordial or medicine. I saw him again next day. He informed me that the pain returned with violence in the evening; but that the application of cold water soon rendered it tolerable, and that he had enjoyed some sleep at intervals during the night. He had taken his breakfast with an appetite. The joints were still swelled but not preternaturally hot, and as he still felt a slight gnawing pain in them, I advised that *tepid* instead of cold water should now be applied. This was immediately done, with great comfort to his feelings. As he had had no alvine motion for two days a laxative was ordered, which I saw taken; and I advised him to apply tepid or cold water to the joints, according as the *temperature* and *sensation of heat* in them should indicate. On visiting my patient next day, he informed me the laxative had produced two motions, that he had only once had occasion to apply the cold water since I saw him, that he had slept well during the night and was now free from complaint, some stiffness in the affected joints excepted. From this time he had no return of the pain; by proper means the stiffness of the joints soon went off, and he thought himself in every respect better, although the paroxysm had been much shorter than usual.

It cannot but appear of considerable importance to ascertain whether or not the gouty actions in the joints were less or greater in this paroxysm, in which the patient had felt so little pain, than in those former ones in which the pain was allowed to continue, and which had been protracted to a much greater length, with a less perfect recovery. When gout is repelled either by excess of heat or of cold, the only symptoms by which the repulsion can be recognized, that I know of, are either the pain being transferred to other joints, or symptoms of increased or of interrupted actions affecting some internal organ, such as diarrhœa, or vomiting, sickness, spasmodic pains, coldness, &c. But in the above case none of these supervened. On the contrary, the sickness and spasms which prevailed while the pained joints were kept hot, gradually and spontaneously subsided after the cold application applications had been continued only for a short time. This would rather seem to indicate that the gouty actions had been increased in the joints, than that they had been repelled from them by the cold applications. This conclusion appears to be further supported by the shortening of the paroxysm and quicker restoration to health; a fact that shews, that however peculiar and different from other diseased actions those of gout may be, they follow the same laws as the plastic actions above treated of, being, cæteris paribus, soonest accomplished when they are not attended with pain.

Physicians of the greatest judgment and experience agree in the opinion, that the only probable means of obtaining a safe and permanent cure of gout, is by attention to avoid the exciting causes. But *when the gouty actions are excited*, it is doubtful if an attempt to check or extinguish them will be attended with either more success or safety, than an attempt to check or extinguish the peculiar actions in small-pox, scarlatina, &c. after they are excited. But when the powers of life are oppressed, and the actions excited opposed, either by excess or defect of heat, or other ascertainable cause; surely, both reason, experience and analogy, vindicate that practice, which by removing these causes enables the diseased actions to run their course with the greatest quickness, with the most comfort and safety to the patient, and with the least derangement to the important functions of the body.

(*To be continued.*)

